# miRNA Stability in Frozen Plasma Samples

**DOI:** 10.3390/molecules201019030

**Published:** 2015-10-20

**Authors:** Francesca Balzano, Marta Deiana, Silvia Dei Giudici, Annalisa Oggiano, Angela Baralla, Sara Pasella, Andrea Mannu, Mario Pescatori, Baingio Porcu, Giuseppe Fanciulli, Angelo Zinellu, Ciriaco Carru, Luca Deiana

**Affiliations:** 1 Department of Biomedical Sciences, University of Sassari, vl. San Pietro 43b, Sassari 07100, Italy; E-Mails: mariafrancesca22@virgilio.it (F.B.); angela.b3@virgilio.it (A.B.); sarapasella18@gmail.com (S.P.); amannu@tiscali.it (A.M.); mariopescatori@gmail.com (M.P.); baingio@gmail.com (B.P.); azinellu@uniss.it (A.Z.); carru@uniss.it (C.C.); 2 Associazione “L’Isola dei Centenari”, Via Milano 4, Sassari 07100, Italy; E-Mail: isoladeicentenari@gmail.com; 3 Istituto Zooprofilattico Sperimentale della Sardegna, Via Vienna 2, Sassari 07100, Italy; E-Mails: silvia.deigiudici@izs-sardegna.it (S.D.G.); annalisa.oggiano@izs-sardegna.it (A.O.); 4 Department of Clinical and Experimental Medicine, University of Sassari, vl. San Pietro 8, Sassari 07100, Italy; E-Mail: gfanciu@uniss.it

**Keywords:** miRNA, stability, long freezing times, sequences AU or UA

## Abstract

MicroRNAs (miRNAs) represent a family of small non-coding ribonucleic acids that post-transcriptionally inhibits the expression of their target messenger RNAs (mRNAs), thereby acting as general gene repressors. In this study we examined the relative quantity and stability of miRNA subjected to a long period of freezing; we compared the stability of eight miRNAs in the plasma of five human healthy controls before freezing and after six and 12 months of storage at −80 °C. In addition, we examined the plasma frozen for 14 years and the amount of miRNA still available. Using a Life Technologies protocol to amplify and quantify plasma miRNAs from EDTA (Ethylene Diamine Tetraacetic Acid)-treated blood, we analyzed the stability of eight miRNAs, (miR-125b-5p, miR-425-5p, miR-200b-5p, miR-200c-3p, miR-579-3p, miR-212-3p, miR-126-3p, and miR-21-5p). The miRNAs analyzed showed a high stability and long frozen half-life.

## 1. Introduction

MicroRNAs (miRNAs) are 20–22-nucleotide-long, non-coding RNA molecules that post-transcriptionally regulate gene expression by base-pairing with the 3′ untranslated region of complementary messenger RNA targets [1,2]. The miRNA is associated with the RNA-induced silencing complex (RISC) [3] and this complex binds target mRNAs through the complementary perfect region seed and partially complementary sequences tail and reduces their translation and/or stability [4]. They regulate diverse biological processes, and bioinformatics data indicate that each miRNA can control hundreds of gene targets, underscoring the potential influence of miRNAs on almost every genetic pathway [5,6,7,8,9,10]. Growing evidence indicates that miRNAs exist not only in cells, but also in a variety of body fluids including blood [11]. Extracellular miRNAs are easy to detect in body fluids, and they have been considered as potential biomarkers for specific diseases [12]. The first studies revealed that these circulating miRNAs may be delivered to recipient cells where they can regulate translation of target genes [13,14]. While many studies have focused on the study of miRNA expression in physiological and pathological processes, various technical problems related to miRNA isolation have simultaneously emerged and the stability of the storage of miRNA in biological samples has been questioned [13,14]. The miRNAs in plasma can be quantitatively detected by methods such as real-time PCR and microarrays [14]. Here we investigate the stability of miRNAs isolated from clinically healthy donors, and evaluate the abundance and solidity of miRNAs subjected to a long period of freezing. To assess the stability of the miRNAs, a real-time PCR analysis was performed on a panel of eight miRNAs from freshly isolated plasma samples and from samples subjected to six and 12 months of storage at −80 °C. Surprisingly, the expression of the tested miRNAs was stable for six and 12 months at −80 °C. miRNAs isolated from stored samples did not show any significant degradation. Moreover, the real-time PCR analysis was repeatedly performed on plasma samples stored over a period of ~14 years. The results showed that the samples were stable for four years and then began to decrease, remaining detectable. All samples were recruited from the biobank of the longevity AKeA Project (project approved by the local Ethics committee) [15,16].

## 2. Results and Discussion

**Figure 1:**
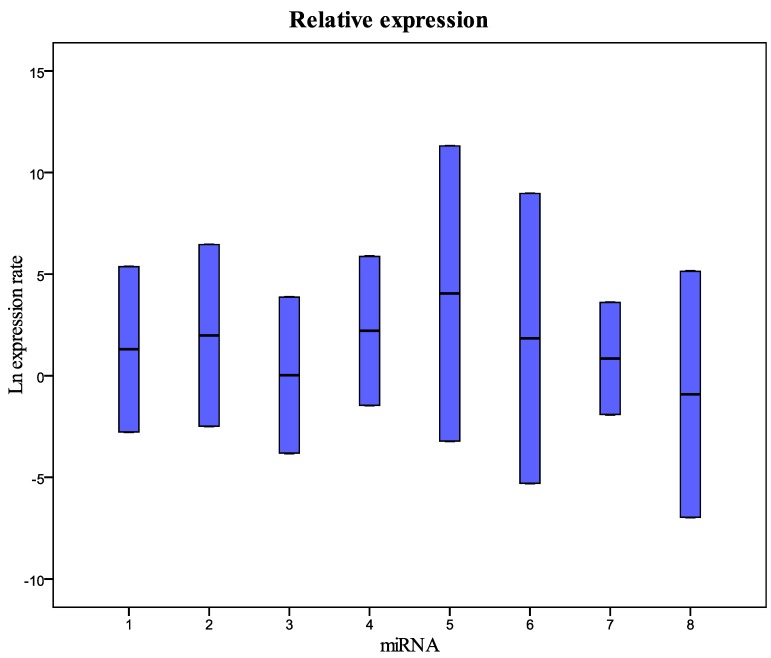
Relative expression (concentration) of the miRNAs analyzed in this study in samples frozen for 12 months compared with fresh samples. 1 = miR-125b-5p; 2 = miR-425-5p; 3 = miR-200b-5p; 4 = miR-200c-3p; 5 = miR-579-3p; 6 = miR-212-3p; 7 = miR-126-3p; 8 = miR-21-5p.

**Figure 2:**
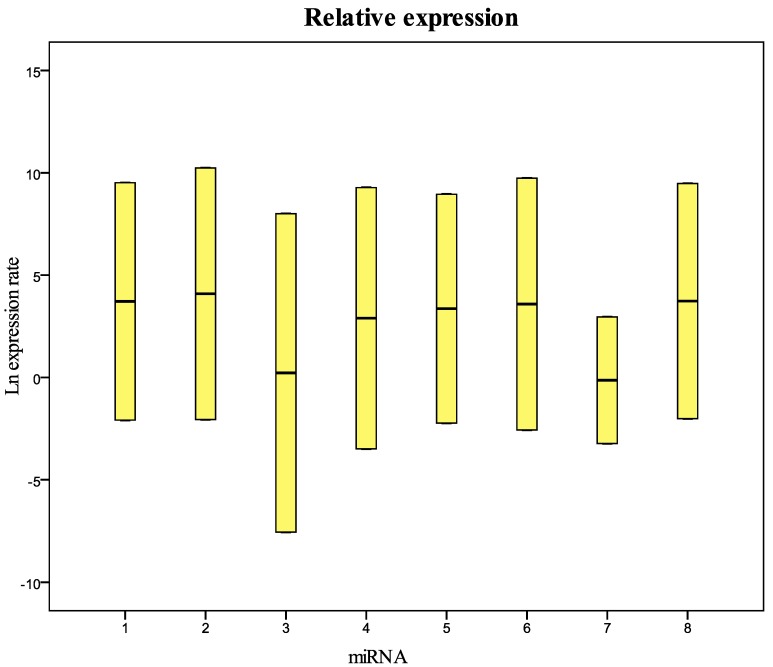
Relative expression (concentration) of the miRNAs analyzed in this study in samples collected in 2010 and frozen at −80 °C compared with fresh samples. 1 = miR-125b-5p; 2 = miR-425-5p; 3 = miR-200b-5p; 4 = miR-200c-3p; 5 = miR-579-3p; 6 = miR-212-3p; 7 = miR-126-3p; 8 = miR-21-5p.

**Figure 3:**
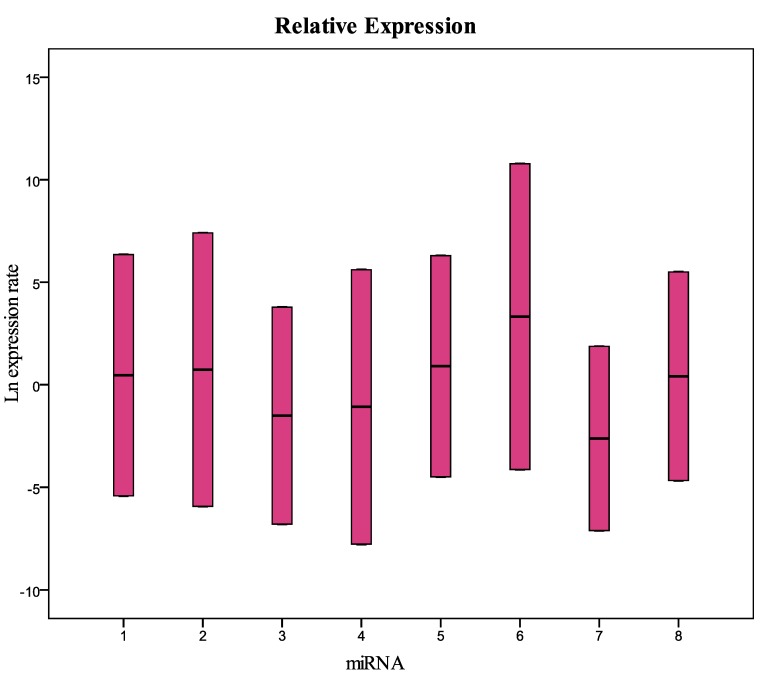
Relative expression (concentration) of the miRNAs analyzed in this study in samples collected in 2009 and frozen at −80 °C compared with fresh samples. 1 = miR-125b-5p; 2 = miR-425-5p; 3 = miR-200b-5p; 4 = miR-200c-3p; 5 = miR-579-3p; 6 = miR-212-3p; 7 = miR-126-3p; 8 = miR-21-5p.

**Figure 4:**
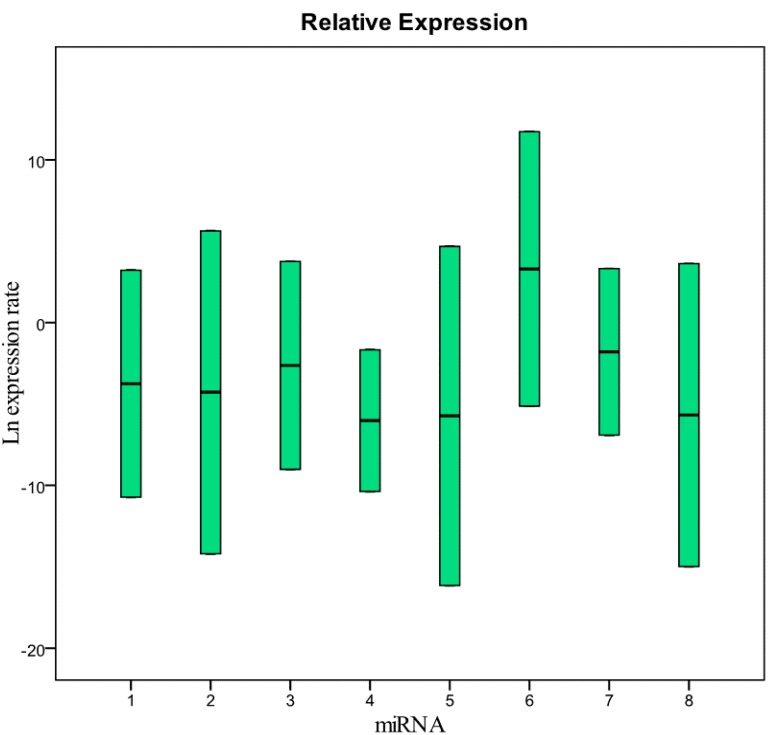
Relative expression (concentration) of the miRNAs analyzed in this study in samples collected in 1999 and frozen at −80 °C compared with fresh samples. 1 = miR-125b-5p; 2 = miR-425-5p; 3 = miR-200b-5p; 4 = miR-200c-3p; 5 = miR-579-3p; 6 = miR-212-3p; 7 = miR-126-3p; 8 = miR-21-5p.

The raw values, the mean and standard deviation of Ct (threshold cycle) of miRNAs and U6snRNA analyzed are reported in supplementary Tables S1 and S2. Data for all targets resulted not normally distributed. The Kruskal-Wallis test showed no significant differences between years only for U6snRNA (*p* = 0.977), miR200b-5p (*p* = 0.099), miR-212-3p (*p* = 0.082), and miR579-3p (*p* = 0.079). The statistical analysis supported our decision to use the U6snRNA as normalizer for the real-time PCR analysis because of its greater stability between groups of samples. We analyzed eight miRNAs: miR-125b-5p; miR-425-5p; miR-200b-5p; miR-200c-3p; miR-579-3p; miR-212-3p; miR-126-3p; and miR-21-5p identified with a number from 1 to 8 in all graphics and tables. We performed a comparison between the level of miRNA in fresh plasma samples and the level of miRNA in frozen samples for different storage periods. In particular, the fresh samples were analyzed at the time of collection, and after six and 12 months of storage at −80 °C. miRNA levels in plasma samples collected in 1999, 2002, 2003, 2009, and 2010 and stored at −80 °C were also evaluated and compared to fresh samples. The Ct value of some samples frozen for six or 12 months showed to be less than that of paired fresh samples; this could be explained with technical error in the RNA extraction and/or RT-PCR procedure. Figure 1 and Table 1 show the relative concentration of the miRNAs analyzed in this study in the samples frozen for 12 months at −80 °C compared with fresh samples. As in the samples frozen for six months, these results show that there are no significant differences in the miRNA levels between fresh and frozen samples. The same results were obtained in the comparison between fresh samples and samples collected in 2010 and stored at −80 °C (Figure 2 and Table 2). The comparative analysis with the samples collected in 2002, 2003, and 2009 (Figure 3 and Table 3) showed that miRNA 126-3p (#7) had a concentration significantly lower (*p* = 0.008) than fresh samples, while the other miRNAs showed no significant differences. Finally, in the samples collected in 1999, all miRNAs had a significantly low concentration, except for the miRNA 212-5p (#6) (Figure 4 and Table 4). These results show that the miRNAs decay at different times. During the first four years we did not observe significant differences in the concentration of miRNAs in fresh samples when compared to frozen samples. All miRNAs begin to decline after five years of freezing, while miR-212-3p does not decay and remains stable after 14 years of freezing. All miRNAs studied seem to show differences in stability in relation to the number of the AU or UA motifs in their sequences. We looked for a possible explanation for this result. In 2009, Sethi and Lukiw suggested a decline linked to the number of AU sequences in the miRNAs under investigation in their study [17]. We observed this result in our samples subjected to freezing in the various time intervals. miRNA 212-3p has one AU sequence, all other miRNAs analyzed have from two to five AU sequences. miRNA 126-3p, which has five AU sequences, was the first to decrease its concentration after five years. We think that miRNA stability is related to the absence of AU sequences in seed and tail miRNA regions.

**Table 1:** Relative expression (concentration), standard error, 95% confidence interval (C.I.), and *p*-value of miRNAs analyzed in this study (samples frozen for 12 months compared with fresh samples). *p*-value (≥0.05) shows that there are no significant differences.

miRNA	Type	Reaction Efficiency	Expression	Std. Error	95% C.I.	P(H1)
1	TRG	1	2.210	0.181–16.260	0.080–246.143	0.468
2	TRG	1	2.141	0.388–76.793	0.039–683.002	0.592
3	TRG	1	0.271	0.016–1.566	0.007–38.257	0.331
4	TRG	1	2.039	0.563–22.424	0.051–170.211	0.497
5	TRG	1	6.907	0.377–496.960	0.020–27.857	0.365
6	TRG	1	1.247	0.024–128.740	0.011–4.842	0.951
7	TRG	1	1.211	0.380–6.831	0.043–34.196	0.765
8	TRG	1	0.342	0.061–3.016	0.001–73.104	0.502
U6snRNA	REF	1	1			

**Table 2:** Relative expression (concentration), standard error, 95% confidence interval (C.I.), and *p*-value of miRNAs analyzed in this study (samples collected in 2010 and stored at −80 °C compared with fresh samples). *p*-value (≥0.05) shows that there are no significant differences.

miRNA	Type	Reaction Efficiency	Expression	Std. Error	95% C.I.	P(H1)
1	TRG	1	6.088	0.376–118.479	0.169–5.649	0.300
2	TRG	1	3.430	0.237–96.389	0.132–11.035	0.543
3	TRG	1	0.205	0.001–58.136	0.001–1.239	0.496
4	TRG	1	1.273	0.060–76.914	0.031–4.285	0.915
5	TRG	1	2.900	0.205–55.911	0.125–3.116	0.526
6	TRG	1	7.825	0.441–1.673.136	0.115–14.474	0.315
7	TRG	1	0.373	0.089–2.646	0.050–11.195	0.254
8	TRG	1	3.317	0.210–148.920	0.144–5.319	0.536
U6snRNA	REF	1	1.000			

**Table 3:** Relative expression (concentration), standard error, 95% confidence interval (C.I.), and *p*-value of miRNAs analyzed in this study (samples collected in 2009 and stored at −80 °C compared with fresh samples). miRNA 126b-5p (#7) decreased significantly (*p* = 0.021) while all other miRNAs show no significant differences.

miRNA	Type	Reaction Efficiency	Expression	Std. Error	95% C.I.	P(H1)	Result
1	TRG	1	1.446	0.075–22.084	0.005–399.228	0.762	
2	TRG	1	0.355	0.027–5.061	0.003–131.362	0.418	
3	TRG	1	0.110	0.002–2.424	0.002–79.778	0.120	
4	TRG	1	0.099	0.005–2.367	0.000–36.781	0.065	
5	TRG	1	0.308	0.014–7.950	0.000–156.536	0.468	
6	TRG	1	9.120	0.817–168.704	0.030–18.989	0.159	
7	TRG	1	0.093	0.020–0.479	0.001–5.080	0.008	DOWN
8	TRG	1	1.229	0.104–16.032	0.012–505.934	0.858	
U6snRNA	REF	1	1.000				

**Table 4:** Relative expression (concentration), standard error, 95% confidence interval (C.I.), and *p*-value of miRNAs analyzed in this study (samples collected in 1999 and stored at −80 °C compared with fresh samples). All miRNAs decreased significantly, except miRNA-212-3p (#6) that shows no significant differences.

miRNA	Type	Reaction Efficiency	Expression	Std. Error	95% C.I.	P(H1)	Result
1	TRG	1	0.009	0.000–0.326	0.000–15.647	0.007	DOWN
2	TRG	1	0.010	0.000–0.739	0.000–56.166	0.017	DOWN
3	TRG	1	0.024	0.003–0.372	0.000–10.244	0.008	DOWN
4	TRG	1	0.001	0.000–0.009	0.000–0.145	0.000	DOWN
5	TRG	1	0.008	0.000–0.838	0.000–39.763	0.021	DOWN
6	TRG	1	2.095	0.041–90.056	0.011–2.538	0.652	
7	TRG	1	0.059	0.005–0.882	0.002–10.576	0.007	DOWN
8	TRG	1	0.002	0.000–0.095	0.000–11.618	0.002	DOWN
U6snRNA	REF	1	1.000				

## 3. Experimental Section

### 3.1. Human Plasma Samples

Plasma samples were from subjects aged between 30 and 50 years. The donors were healthy volunteers recruited from the longevity AKeA Project (project approved by the local bioethics committee). Subjects signed a written consent prior to blood sampling. Blood samples were collected early in the morning (to reduce the biological variability) by venipucture into a vacutainer (Greiner Bio-One, Monroe, NC, USA) containing K2 EDTA as an anticoagulant. Then we proceeded to the separation of the plasma by centrifugation at 2500 *g* for 15 min at 4 °C. The supernatant containing the plasma was divided into a defined number of aliquots of 200 µL each. Five samples were collected in 2013 and analyzed directly or after a storage of six and 12 months at −80 °C. The other 24 samples from different donors were from the AkeA Project Biobank, collected in 1999, 2002, 2003, 2009, and 2010, and had been stored at −80 °C. All defrozen samples were carefully mixed before analysis. The samples’ characteristics are shown in Table 5.

**Table 5:** Sample Collection: number, collection year, and status of samples analyzed.

Plasma Samples Number	Sample Status	Collection Year
5	Fresh	2013
5	Stored 6 months at −80 °C	2013
5	Stored 12 months at −80 °C	2013
5	Stored at −80 °C	2010
5	Stored at −80 °C	2009
5	Stored at −80 °C	2003
4	Stored at −80 °C	2002
5	Stored at −80 °C	1999

### 3.2. RNA Extraction

RNA was extracted from plasma fractions using miRNeasy Serum/Plasma Kit (50) (Qiagen, Milan, Italy) according to the manufacturer’s instructions, with the final elution volume of 15 μL. Eight individual miRNAs (miR-125b-5p, miR-425-5p, miR-200b-5p, miR-200c-3p, miR-579-3p, miR-212-3p, miR-126-3p, and miR-21-5p) were selected based on their expression in the plasma of healthy donors and amplification efficiency and studies previously conducted [18,19].

### 3.3. qPCR Analysis

The concentration level of mature miRNAs was tested by quantitative real-time PCR (qPCR) using TaqMan^®^ MicroRNA Reverse Transcription Kit, Life Technologies (Carlsbad, CA, USA), for the reverse transcription. TaqMan^®^ Universal Master Mix II, Life Technologies, was used for the PCR according to the manufacturer’s instructions; 45 amplification cycles were performed. miRNA concentration levels were quantified using the IQ5, BIORAD, instrument (Milan, Italy). The U6snRNA was used for the data normalization [20,21]. Real-time PCR was done in duplicate. The miRNAs analyzed in this study were identified with progressive numbers from 1 to 8 (Table 6). The sequences and the identification symbols were retrieved from miRbase and are reported in Table 6 [22].

**Table 6:** miRNA collection: accession number, symbol, sequence, and identification number used in this study of miRNA analyzed.

Accession	Symbol	Sequence ID Number
IMAT0000423	hsa-miR-125b-5p	UCCCUGAGACCCUAACUUGUGA 1
MIMAT0003393	hsa-miR-425-5p	AAUGACACGAUCACUCCCGUUGA 2
MIMAT0004571	hsa-miR-200b-5p	CAUCUUACUGGGCAGCAUUGGA 3
MIMAT0000617	hsa-miR-200c-3p	UAAUACUGCCGGGUAAUGAUGGA 4
MIMAT0003244	hsa-miR-579-3p	UUCAUUUGGUAUAAACCGCGAUU 5
MIMAT0000269	hsa-miR-212-3p	UAACAGUCUCCAGUCACGGCC 6
MIMAT0000445	hsa-miR-126-3p	UCGUACCGUGAGUAAUAAUGCG 7
MIMAT0000076	hsa-miR-21-5p	UAGCUUAUCAGACUGAUGUUGA 8

### 3.4. Statistical Analysis and Real-Time PCR Data Analysis

The raw Ct values for each miRNA and U6snRNA were checked for normal distribution. The Kruskal-Wallis test was applied to compare the groups (years) in each target. All the analysis and graphics were performed with the SPSS software version 17.0. Reverse transcription followed by polymerase chain reaction (RT-PCR) is the most suitable method for the detection and quantification of miRNA. It provides high sensitivity, good reproducibility, and a wide range quantification. Several mathematical algorithms have been developed to calculate a ratio of expression based on real-time PCR efficiency and the crossing point deviation of an unknown sample against a control. Then a software tool named REST^©^(relative expression software) [23] was used, which compares two groups, with a maximum of 16 data points in a sample and 16 in a control group, for reference and up to four target genes. The mathematical model used is based on the PCR efficiencies and the crossing point average gap between sample and control. Subsequently, the reported expression or concentration of transcripts investigated are tested for significance by a randomization test. The relative concentration of the mature miRNAs was analyzed using the software REST. The non-parametric bootstrapping test was used to evaluate concentration differences of miRNAs between frozen and fresh samples. The five samples collected in 2013 were subjected to paired analysis between time-points (freshly isolated *vs.* stored six or 12 months at −80 °C) while all other samples were compared to fresh samples.

## 4. Conclusions

All miRNAs studied did not show differences in relative abundance between fresh samples and those subjected to four years of long freezing. The comparison between fresh and paired stored samples and the observed technical error highlights the need to optimize the current methods of analysis to improve their consistency. However, miRNA-212-3p showed to be significantly resistant to a long freezing time (14 years). The concentration of the other miRNAs has been proven to decrease after long freezing times, but they were still present, showing an elevated stability. As previously reported, all miRNAs studied seem to show differences in the stability in relation to the number of the sequences containing AU or UA [17]. We suppose that miRNAs’ half-life and their degradation plays an important biological role and that the decline of miRNA is written in its sequence and is modulated by an editing substitution of a cytosine with an uracil during biogenesis. This may explain why in plants there is a perfect pairing of miRNA with the mRNA. In animals, pairing is perfect only in the seed region but not in the extra seed region and this fact can have a relevant role in the modulation of miRNAs’ half-life. In animals, partial pairing between miRNA and the mRNA target site usually results in reduced protein expression through a variety of mechanisms. miRNA function seems to be distinct and less regulated in plants, in which miRNAs perfectly base-pair with targets and induce their degradation [24]. In conclusion, we think that this is a further control mechanism of the biological role played by miRNA. In animals, there is a more refined control of the “half-life” of miRNAs. These considerations could open new development into the utilization of miRNAs as therapeutic drugs.

## Supplementary Materials

Supplementary materials can be accessed at: http://www.mdpi.com/1420-3049/20/10/19030/s1.
