# Supplementary material for: miRNA Stability in Frozen Plasma Samples
Source: Molecules. 2015 Oct 20;20(10):19030–40. doi: 10.3390/molecules201019030 (PMC6331950; doi:10.3390/molecules201019030)
Supplement: Supplementary file 1 [file molecules-20-19030-s001.pdf]

Supplementary Materials

Table S1. Ct values of miRNAs and U6snRNA analyzed in this study.

Year	miR-125b-5p	miR-425-5p	miR-200b-5p	miR-200c-3p	miR-579-3p	miR-212-3p	miR-126-3p	miR-21-5p	U6snRNA
2013	34.98	29.69	37.37	36.24	36.63	30.22	26.82	28.26	38.12
2013	35	29.01	37.64	36	35	30.46	28.11	29.1	38.11
2013	36.72	33.27	37	36.85	36.88	29.83	26.95	29.05	32.89
2013	34.73	31.27	37.13	35.96	35.88	30.18	27.03	29.08	38.9
2013	37.28	29.69	37.09	34.73	34.5	43.02	27.82	27.53	37.9
2013 *	34.56	30.65	39.97	35.45	35.98	30.34	27.21	26.90	38.12
2013 *	33.68	29.45	36.13	34.65	35.12	31.09	27	28.12	37.56
2013 *	33.12	29.78	38.1	34.23	36.54	31.11	26.18	26.89	35.33
2013 *	37.24	30.43	41.15	34.89	25.16	36.34	27.21	37.10	37.45
2013 *	35.12	29.58	37.89	33.99	32.98	33.76	27.37	29.98	38.47
2013 **	34.13	30.03	39.89	35	35.76	30.61	27.2	27.88	38
2013 **	33.97	29.59	36.91	34.27	35.14	31.09	27.11	28.03	38
2013 **	33.59	29.63	37.66	34.87	36.02	31	26.64	27	33.61
2013 **	37.2	29.09	44.1	35.4	25.93	36.32	27.3	37.75	37.98
2013 **	34	29	37	35	32	33	27	30	38.23
2010	35.3	30.94	37.89	38.95	36.25	30.28	29.42	29.62	37.12
2010	28.14	23.65	30.6	28.61	29.11	29.11	29.6	20.52	38
2010	34.7	29.73	45	37.72	35.68	30.85	28.65	28.28	35.9
2010	35.09	31.17	39.4	36.85	35.97	30.34	28.73	28.67	38.9
2010	34.35	30.45	46.66	37.81	36.1	30.19	29.34	29.18	37.9
2009	33.93	30.65	36.73	36.61	37.14	28.92	28.51	28.2	37.11
2009	34.21	32.75	45	45	35.91	28.7	30.25	30.09	37
2009	35.61	31.42	39.53	35.72	35.04	29.2	30.35	28.33	32
2009	33.6	28.63	37.59	34.8	33.84	29.64	30.46	27.16	38.9
2009	32.83	31.19	45	38.72	35.48	27.5	30.61	28.61	37.9
2003	37.32	32.39	38.76	45	36.73	26.44	31.85	32.68	38.16
2003	35	38.34	45	45	35.89	31.34	30.95	37.89	33.67
2003	45	45	45	45	45	32.9	30.87	40.56	38.91
2003	37.56	31.88	45	38	35.87	32.41	27.91	34.55	33.6
2003	35.38	31.39	37.84	37.12	37.65	23.39	30.55	30.81	38.56
2002	37.20	32.09	46.39	35.40	35	36.32	27.30	37.75	38.54
2002	37.32	32.38	30.84	45	36.73	26.33	31.88	32.68	33.76
2002	32.83	31.19	45	38.72	35.48	27.50	28.74	28.61	34.89
2002	33.64	30.40	45	45	45	28.69	27.74	28.18	38.12
1999	45	37.69	45	45	45	36.94	33.92	42.52	38.3
1999	45	38.11	45	45	45	31.81	31.79	38.69	38
1999	45	45	45	45	45	30.97	31.87	45	33.8
1999	38.13	30.88	45	45	35.87	32.41	27.91	34.55	38.6
1999	37.32	32.38	38.84	45	36.73	26.33	31.88	32.68	38.1

* Stored 6 months at −80 °C; ** Stored 12 months at −80 °C. Ct values > 45 or undetermined were replaced with 45.

Table S2. Mean and standard deviation of Ct of miRNAs and U6snRNA analyzed in this study.

Year	miR-125b-5p	miR-425-5p	miR-200b-5p	miR-200c-3p	miR-579-3p	miR-212-3p	miR-126-3p	miR-21-5p	U6snRNA
2013	35.74 ± 1.17	30.59 ± 1.71	37.25 ± 0.26	35.96 ± 0.77	35.78 ± 1.02	32.74 ± 5.75	27.35 ± 0.6	28.60 ± 0.7	37.18 ± 2.4
2013 *	34.74 ± 1.59	29.98 ± 0.53	38.65 ± 1.95	34.65 ± 0.57	33.16 ± 4.67	32.52 ± 2.50	26.99 ± 0.47	29.80 ± 4.27	37.39 ± 1.22
2013 **	34.58 ± 1.48	29.47 ± 0.42	39.11 ± 3.04	34.91 ± 0.41	32.97 ± 4.25	32.40 ± 2.38	27.05 ± 0.25	30.13 ± 4.40	37.16 ± 1.99
2010	33.52 ± 3.02	29.19 ± 3.14	39.91 ± 6.37	35.99 ± 4.19	34.62 ± 3.09	30.15 ± 0.64	29.15 ± 0.43	27.25 ± 3.80	37.56 ± 1.12
2009	34.04 ± 1.02	30.93 ± 1.50	40.77 ± 3.99	38.17 ± 4.08	35.48 ± 1.21	28.79 ± 0.80	30.04 ± 0.86	28.48 ± 1.05	36.58 ± 2.67
2003	38.05 ± 4.05	35.8 ± 5.86	42.32 ± 3.68	42.02 ± 4.09	38.23 ± 3.85	29.30 ± 4.18	30.43 ± 1.49	35.30 ± 3.93	36.58 ± 2.70
2002	35.24 ± 2.35	31.5 ± 0.90	41.81 ± 7.34	41.03 ± 4.78	38.05 ± 4.69	29.71 ± 4.51	28.91 ± 2.06	31.80 ± 4.45	36.32 ± 2.36
1999	42.09 ± 3.99	36.81 ± 5.57	43.77 ± 2.75	45 ± 0	41.52 ± 4.77	31.69 ± 3.79	31.47 ± 2.18	38.69 ± 5.19	37.36 ± 2.00
Total	36.02 ± 4.35	31.79 ± 4.79	40.41 ± 4.74	38.40 ± 4.73	36.18 ± 4.21	30.94 ± 3.07	29.92 ± 1.67	31.24 ± 5.68	37.03 ± 2.10

* Stored 6 months at −80 °C; ** Stored 12 months at −80 °C.
